# Promotion of Intestinal Peristalsis by *Bifidobacterium* spp. Capable of Hydrolysing Sennosides in Mice

**DOI:** 10.1371/journal.pone.0031700

**Published:** 2012-02-22

**Authors:** Mitsuharu Matsumoto, Atsushi Ishige, Yuka Yazawa, Manami Kondo, Koji Muramatsu, Kenji Watanabe

**Affiliations:** 1 Dairy Science and Technology Institute, Kyodo Milk Industry Co. Ltd., Hinode-machi, Tokyo, Japan; 2 Laboratory of Kampo Pharmacology, School of Pharmacy, Yokohama College of Pharmacy, Yokohama, Kanagawa, Japan; 3 Analytical Research Center, Kyodo Milk Industry Co. Ltd., Hinode-machi, Tokyo, Japan; 4 Center for Kampo Medicine, School of Medicine, Keio University, Shinjuku-ku, Tokyo, Japan; East Carolina University School of Medicine, United States of America

## Abstract

**Background:**

While there are a variety of identifiable causes of constipation, even idiopathic constipation has different possible mechanisms. Sennosides, the main laxative constituents of *Daio*, an ancient Kampo medicine, are prodrugs that are converted to an active principle, rheinanthrone, by intestinal microbiota. In this study, we aimed to determine the sennoside hydrolysis ability of lactic acid bacterial strains and bifidobacteria in the intestine and to investigate their effect on intestinal peristalsis in mice.

**Methodology/Principal Findings:**

A total of 88 lactic acid bacterial strains and 47 bifidobacterial strains were evaluated for their ability to hydrolyze sennosides. Our results revealed that 4 strains, all belonging to the genus *Bifidobacterium,* had strong sennoside hydrolysis ability, exhibiting a decrease of >70% of sennoside content. By thin-layer chromatography analysis, rheinanthrone was detected in the medium cultured with *B. pseudocatenulatum* LKM10070 and *B. animalis* subsp. *lactis* LKM512. The fecal sennoside contents significantly (*P*<0.001) decreased upon oral administration of these strains as compared with the control. Intestinal peristalsis activity was measured by the moved distance of the charcoal powder administered orally. The distance travelled by the charcoal powder in LKM512-treated mice was significantly longer than that of control (*P*<0.05). Intestinal microbiota were analysed by real-time PCR and terminal-restriction fragment length polymorphism. The diversity of the intestinal microbiota was reduced by kanamycin treatment and the diversity was not recovered by LKM512 treatment.

**Conclusion/Significance:**

We demonstrated that intestinal peristalsis was promoted by rheinanthrone produced by hydrolysis of sennoside by strain LKM512 and LKM10070.

## Introduction

Constipation is a common clinical problem that can be difficult to manage. It has a variety of identifiable causes, but even idiopathic constipation has different possible mechanisms. Chronic idiopathic constipation (CIC) is a common condition, with a prevalence of 4% to 20% in cross-sectional community-based surveys in developed countries [1]–[5]. Constipation is both a symptom and, when chronic, a multisymptom disorder, and it can overlap with other gastrointestinal tract disorders such as dyspepsia and gastro-esophageal reflux disease. Furthermore, one should keep in mind the possibility of cancer and be alert for its warning signs [6]. Therefore, in the last 10 years, novel drug therapies have been developed for constipation, particularly for CIC [7]. Since ancient times, Kampo medicine *daio* (*Rhei rhizoma* or *Sennae folium*) has been employed as a purgative crude drug because of its purgative properties. Sennoside A and B, the main laxative constituent of *daio*, are prodrugs that are converted to an active principle, rheinanthrone, by intestinal microbiota [8]–[11]. Therefore, there is a considerable difference in the efficacy of this drug among individuals. Further, if the patient's intestinal microbiota cannot hydrolyse sennoside A and/or B to rheinanthrone, this treatment would be ineffective. In contrast, certain patients experience diarrhoea as a side effect of *daio* medicine. Since 1970, several studies have been performed to investigate the relationship between sennosides and intestinal microbiota and the metabolism of sennosides [8], [12], [13]. The bacterial strains that can hydrolyse sennosides were isolated from intestines [14]–[16], and these microbiota have been found to employ several pathways to metabolize sennosides [13]. However, to the best of our knowledge, no study has been conducted with regard to the effect of *daio*/sennosides in combination with intestinal microbiota on intestinal peristalsis.

In this study, we used lactic acid bacteria and *Bifidobacterium* spp. isolated from fermented milk and human feces, screened them for their ability for sennoside hydrolysis and investigated their effect in combination with the strains on intestinal peristalsis in mice.

## Materials and Methods

### Bacterial strains

A total of 88 lactic acid bacterial strains belonging to the genera *Lactobacillus*, *Leuconostoc*, *Lactococcus*, *Enterococcus*, and *Streptococcus* were used in this study. Of these, 82 strains were isolated from several fermented milk samples from all parts of the world, 3 were industrial starter cultures used for the manufacture of fermented milk, and 3 were obtained from the Japan Collection of Microorganisms (JCM; RIKEN, Saitama, Japan). A total of 47 bifidobacterial strains belonged to the species *Bifidobacterium adolescentis*, *B. longum*, *B. breve*, *B. bifidum*, *B. catenulatum*, *B. pseudocatenulatum*, and *B. animalis* subsp. *lactis*, including strains isolated from the feces of healthy adults [17]; 1 was a probiotic strain with many health-promoting effects; and 4 were obtained from the JCM. These bacterial strains were isolated in another study performed in 2,000, more than 10 years ago [17]. We obtained permission to stock and use isolated strains in the future from volunteers enrolled to the old study in 2,000.

Strains of lactic acid bacteria were pre-incubated aerobically at 37°C for 48 hours in Lactobacilli MRS broth (Becton Dickinson) or GYP agar [18]. *Bifidobacterium* strains were pre-incubated on blood-liver (BL) agar (Nissui Pharmaceutical, Tokyo, Japan) at 37°C for 72 hours under anaerobic conditions with the aid of an AnaeroPack Anaero system (Mitsubishi Gas Chemical, Tokyo, Japan).

### Assay of sennosides hydrolysis by bacterial strains

Sennoside A or B (Wako) was added to MRS or GYP liquid medium (final concentration: 50 µg/ml) and sterilized by filtration. The medium was added into the wells of a 24-well plate (Nunc), and one bacterial colony on the pre-cultured plate was inoculated in the wells and cultured at 37°C for 48 hours under anaerobic conditions using the AnaeroPack Anaero system. After cultivation, the culture medium was centrifuged at 12,000× *g* for 10 minutes, and the supernatant was stored at −80°C until use. Areas of Sennoside A and B in the supernatant of the media before and after cultivation were quantified by high-performance liquid chromatography (HPLC), and the sennoside hydrolysis rates were determined from the results.

### HPLC conditions

HPLC separation was conducted on a Waters Alliance 2696 system (Waters, MA, USA) equipped with an autosampler, a temperature-controlled column, and Waters 2487 Dual Absorbance Detector working at 340 nm. Sennosides were separated on a Inertsil ODS-3 column (3 mm I.D. ×250 mm, particle size 4 µm, GL Science, CA, USA). The mobile phase consisted of acetonitrile/phosphoric acid. The gradient profiles were as follows: 0 min 14:86, 10 min 14:86, 28 min 35:65, 29 min 80:20, 34 min 80:20. The flow-rate was 0.8 ml/min. The column temperature was 50°C.

### Thin-layer chromatography analysis for rheinanthrone in culture medium

The bacterial strains were added to sennoside-containing culture broth and cultured at 37°C for 35 hours. At the end of the incubation period, the growth was arrested by adding 1% *p*-nitroso-*N,N*′-dimethylaniline. After being maintained at room temperature for 10 min, the mixture was centrifuged at 16,000 *g* for 5 min. Chloroform (1 ml) was then added to the supernatant (2 ml), the mixture was vigorously shaken, and centrifuged again at 300 *g* for 10 min. The chloroform layer was removed and filtered [15]. Aliquots (5 µl) were loaded onto silica gel 60 (Merck), which was developed using *n*-propanol∶ethyl acetate∶water (40∶40∶30) as the solvent [19]. After drying for 30 min, rheinanthrone was visualised by spraying with ammonia solution. The rheinanthrone spot on the silica gel was visualised as a yellow-coloured spot, and its Rf value was measured.

### Mice

Male and female 12- to 15-week-old Crj:CD-1 (ICR) mice bred from same parents (first delivery: 15 weeks, second delivery: 25 weeks) obtained from Japan SLC Inc. (Hamamatsu, Japan) were used. Plastic cages containing hardwood bedding were used (225×338×140 mm^3^; Clea Japan, Inc. Tokyo, Japan); 5 mice were housed per cage. The mice were kept on a 12-hour light/dark cycle at 25±1°C in conventional conditions. They were provided with a standard pellet chow diet (CRF-1, Oriental Yeast Co. Ltd., Tokyo, Japan) *ad libitum.* All animal experiments were approved by the Kyodo Milk Animal Use Committee (Permit Number: 2008-03) and were in accordance with the Guide for the Care and Use of Laboratory Animals, published by the National Academies Press.

### Sennoside hydrolysis assay in mouse intestine by *Bifidobacterium* strains

**Figure 1:**
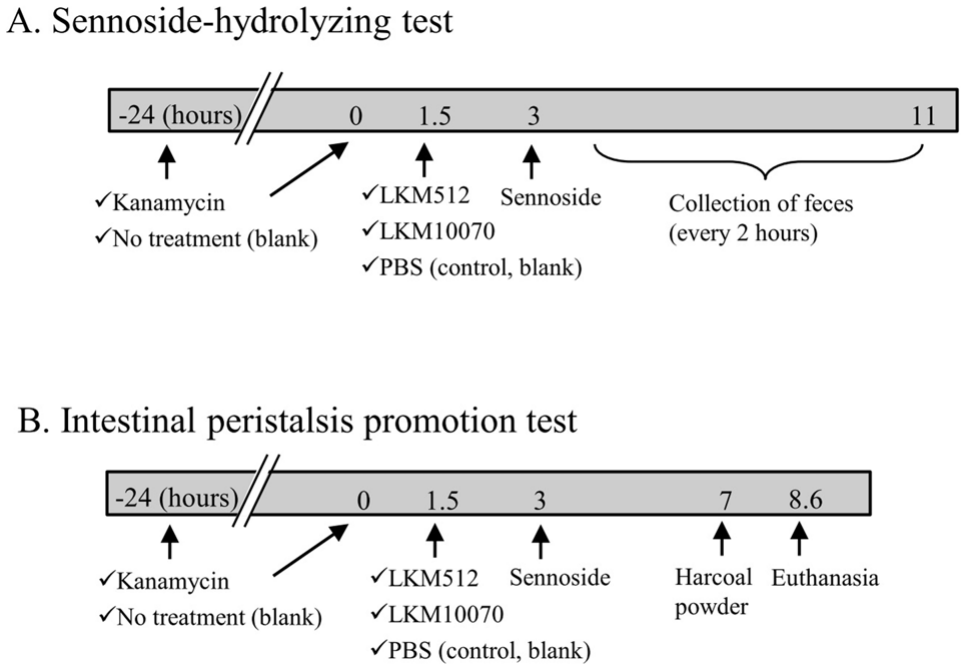
Time schedule of animal assay. These tests were performed individually (one mouse/cage). (A) Sennoside-hydrolysing test by *Bifidobacterium* spp. Feces were collected every 2 hours. (B) Intestinal peristalsis promotion test by LKM512. This test was performed using a pair of mice: 1 LKM512-administered mouse and 1 control mouse.

The timeline of the assay is presented in Figure 1A. Kanamycin was orally administered to the mice 2 times for 2 days in order to damage their normal intestinal bacteria (75 mg/d per mouse; Wako, Tokyo, Japan), because the normal intestinal microbiota of mice can adequately hydrolyse sennosides. Kanamycin was the antibiotic chosen because it is not absorbed from the intestine by the body [20]. *B. pseudocatenulatum* LKM10070 (initially designated as strain M8 during the screening) and *B. animalis* subsp. *lactis* LKM512 solutions were harvested from BL agar containing the cell cultures by swabbing and suspending the swabbed culture in phosphate-buffered saline (PBS). Ninety minutes after the last administration of kanamycin, the mice were orally administered these solutions (0.4 ml/mouse; 5×10^8^–1×10^9^ cfu/mouse). To avoid any genetic variations, 8 males and 9 females born from the same parents were administered *B. pseudocatenulatum* LKM10070 and *B. animalis* subsp. *lactis* LKM512, respectively. Ninety minutes after administration, the mice were orally administered sennoside A dissolved in sodium hydrogen carbonate (0.75 mg/mouse). Thereafter, feces discharged were collected every 2 hours for up to 8 hours and stored at −20°C. Control mice were administered PBS without bacterial cells. The blank test was performed without kanamycin administration. Briefly the mice were treated with PBS not containing bacterial cells but did not receive kanamycin.

From the feces, sennoside A was extracted by shaking the obtained feces sample in 5 ml methanol (70%) for 30 min. After extraction, the supernatant was obtained by centrifugation at 1,500 *g* for 10 min, and the precipitate was extracted again using the same method. The supernatants were mixed, filtered (0.45 µm), and stored at −20°C until sennoside mesurement.

### Animal assay of intestinal peristalsis

The timeline of the assay is presented in Figure 1B. This assay was performed using 10 male mice and *B. animalis* subsp. *lactis* LKM512. The mice were orally administered kanamycin, LKM512 or PBS (control), and sennoside A in order to assay sennoside hydrolysis in the intestine by *Bifidobacterium* strains. After 4 hours, the mice were orally administered 0.4 ml of 5% charcoal powder suspended in 10% gum arabic solution. After 100 minutes, the mice were sacrificed using diethyl ether, and the gastrointestinal tracts were obtained. Intestinal peristalsis activity was expressed as the distance of the charcoal powder from the end of the ileum (i.e. the base of the caecum or start of the colon) to the start of the charcoal powder measured using a ruler. Each pair of mice, of which one was administered LKM512 and the other was used as the control, was assayed simultaneously, and each pair was assayed one after the other because of the exact time line required to be followed during the assay.

### Real-time PCR for quantitative determination of bacterial cell numbers

Fecal specimens were collected from the mice before the treatment, after kanamycin administration (1.5 hours after the last dose of kanamycin), and 3 and 6 hours after LKM512 administration. Bacterial DNA was isolated from 20 mg feces using the methods described by Matsuki et al. [21]. For obtaining the total bacterial count and *B. animalis* subsp. *lactis* count, real-time PCR was performed using the StepOne real-time PCR system (Applied Biosystems, CA, USA) as described in our previous report [22], with minor modification. Briefly, SYBR® *Premix Ex Taq*™ II was used instead of SYBR® *Premix Ex Taq.* The primer sets used are shown in Supplementary Table S1.

### Comparison of fecal microbiota using terminal restriction fragment length polymorphism (T-RFLP)

T-RFLP analysis using 529F primer labelled at the 5′-end with 6-carboxyfluorescein was performed as described in our previous report [22] with some modifications. Briefly, 50 ng of the purified PCR products was digested with 0.25 units of *Hae*III (Takara) at 37°C for 3 hours. The PCR products and the restriction digest products were purified by GenElute PCR Clean-Up Kit (Sigma). The terminal-restriction fragment (T-RF) length was determined using a 1200LIZ standard size marker (Applied Biosystems) in a 3130 Genetic Analyzer (Applied Biosystems) and with the aid of Gene Mapper software v4.0 (Applied Biosystems).

### Statistical analysis

The fecal sennoside contents were compared using Mann-Whitney *U* tests. The distance travelled by the charcoal powder measured in the intestinal peristalsis assay was compared using a modified Wilcoxon signed-rank test. StatMate IV (ATMS Co. Ltd., Tokyo, Japan) was used to conduct all the statistical analyses.

## Results

### Screening of sennoside-hydrolysing bacterial strains

**Figure 2:**
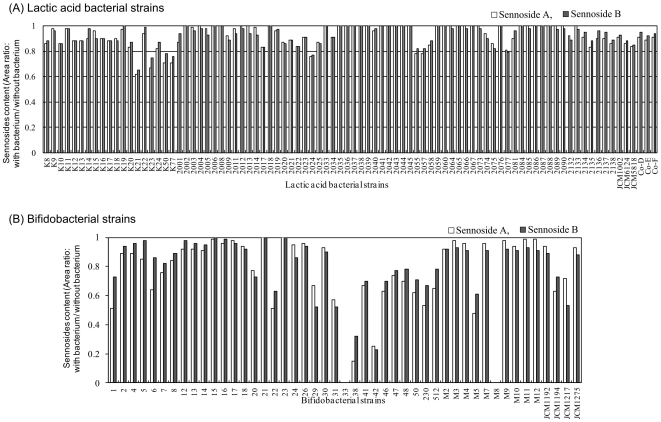
Ability of lactic acid bacterial strains and bifidobacterial strains to hydrolyse Sennoside A and B. Sennoside-hydrolysing values were expressed as the ratio of sennoside content in the medium without bacterial strain to that in the medium with bacterial strain.

The quantity of sennoside A and B in the medium after cultivation was expressed as area ratio (culture with bacterium/culture without bacterium). In 5 of the 88 lactic acid bacterial strains (Figure 2A) and 21 of the 47 bifidobacterial strains (Figure 2B), the sennoside content of the medium had decreased over 20%, suggesting that these strains have the ability to hydrolyse sennosides. Specifically, *Bifidobacterium* strains 33, 38, 42, and M8 had strong sennoside hydrolysis ability; in these strains, a decrease of >70% of sennoside content was observed. After screening these 4 strains, we selected *B. pseudocatenulatum* M8 (hereafter referred to as LKM10070; the 16S rRNA sequence of this strain was read and renamed for the stock) because it had the strongest acid tolerance among all the 4 strains (data not shown). We also selected *B. animalis* subsp. *lactis* LKM512 due to its moderate ability of sennoside hydrolysis. While LKM10070 hydrolyses sennoside rapidly, LKM512 hydrolyses it at a slow rate. LKM512 exhibits potent acid tolerance [23] and the ability to adhere to human intestinal mucin [24], and it alters the intestinal microbial populations [25]. The bacterial counts of both LKM10070 and LKM512 in the liquid medium after cultivation were 10^9^ cfu/ml.

**Figure 3:**
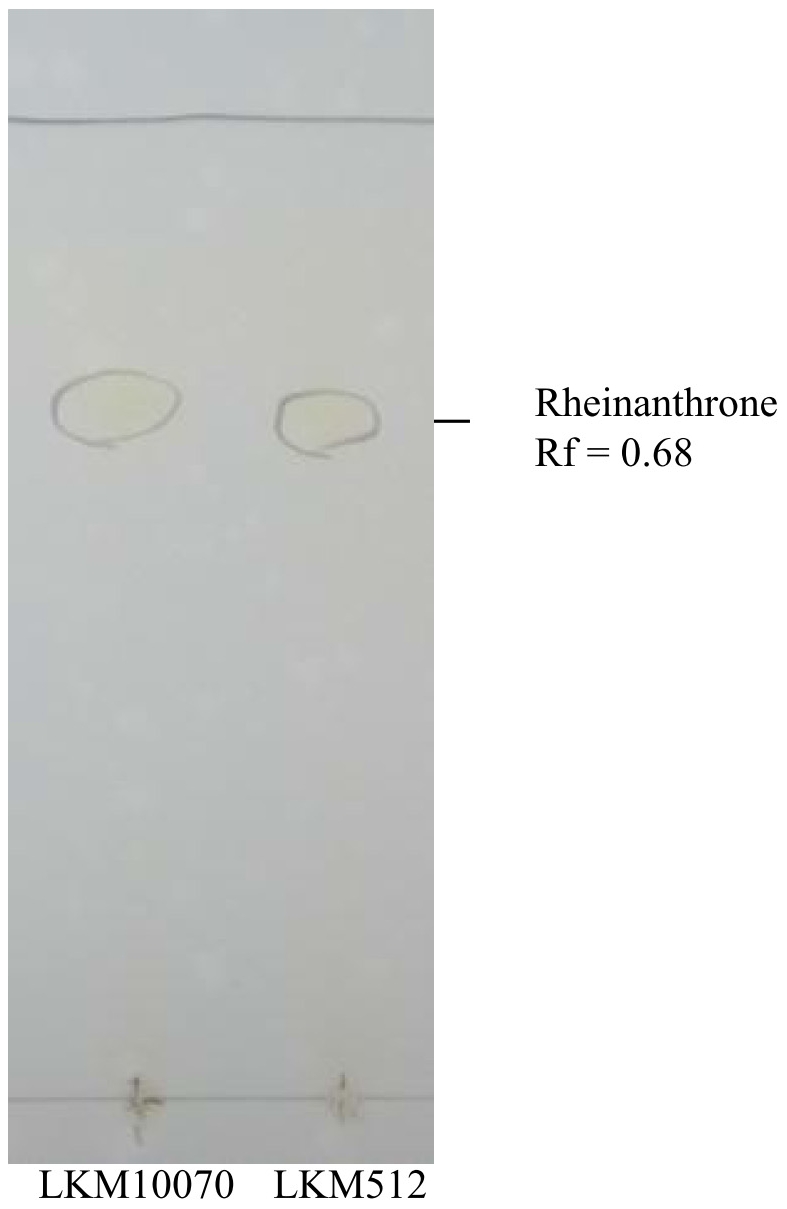
Detection of rheinanthrone in sennoside-containing medium cultivated by bifidobacterial strain LKM10070 or LKM512 by thin-layer chromatography (TLC). Spots of rheinanthrone were coloured yellow, and the Rf value was 0.68.

By thin-layer chromatography (TLC), rheinanthrone was detected as yellow spots with Rf values of 0.68 in the medium cultured with LKM10070 or LKM512; this value was almost the same as the reference value 0.64 (Figure 3) [19].

### Sennosides hydrolysis in the intestine by *Bifidobacterium* strains

**Figure 4:**
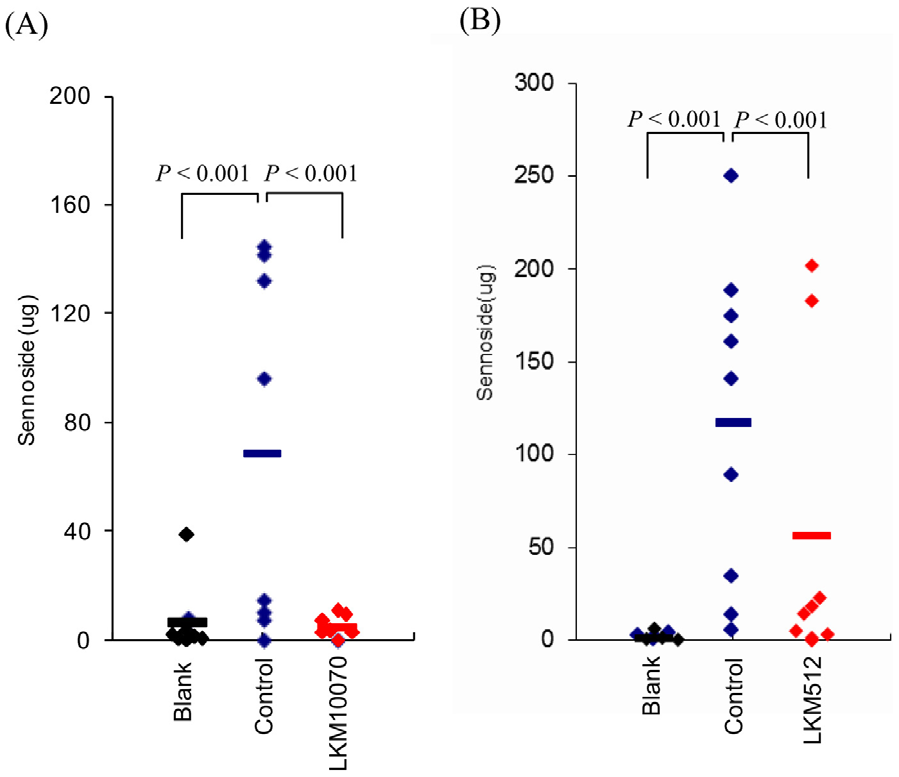
Sennoside hydrolysis in the intestine by bifidobacterial strain LKM10070 (A) and LKM512 (B) administered orally after kanamycin treatment. Feces were collected every 2 hours up to 8 hours. The total sennoside contents in feces discharged up to 8 hours after administration of sennoside were significantly higher in kanamycin-treated mice than in the blank (no kanamycin) (*p*<0.001). These tests for the blank group were performed before kanamycin treatment. The amount of sennoside collected from the feces of mice administered LKM10070 (n = 8) or LKM512 (n = 9) was significantly lower than that collected from the feces of control mice (*p*<0.001, Mann-Whitney *U* test). Bars indicate the average value. The times of dosages are shown in Materials and Methods section and in Figure 1.

The total sennoside contents in feces discharged up to 8 hours after sennoside administration were 2.2±1.1, 117.5±28.7, and 56.3±28.2 µg in the blank, control, and LKM10070 group, respectively. Levels in the LKM10070 group were significantly decreased as compared with the blank (*p*<0.001) and the control (*p*<0.001) groups (Figure 4A). The total sennoside contents in feces discharged up to 8 hours after sennoside administration in the blank, control, and LKM512 group were 6.6±4.1, 68.4±22.1, and 4.4±1.3 µg, respectively. Levels in the LKM512 group were significantly decreased as compared with the blank (*p*<0.001) and the control (*p*<0.001) groups (Figure 4B). These results indicate that bifidobacterial strains hydrolyzed sennoside in the intestine (Figure 4). Mice in which the fecal sennoside content decreased discharged loose feces; further, almost all the mice (7 out of 8 mice) treated with LKM10070 had diarrhea.

### Promotion of intestinal peristalsis by LKM512

**Figure 5:**
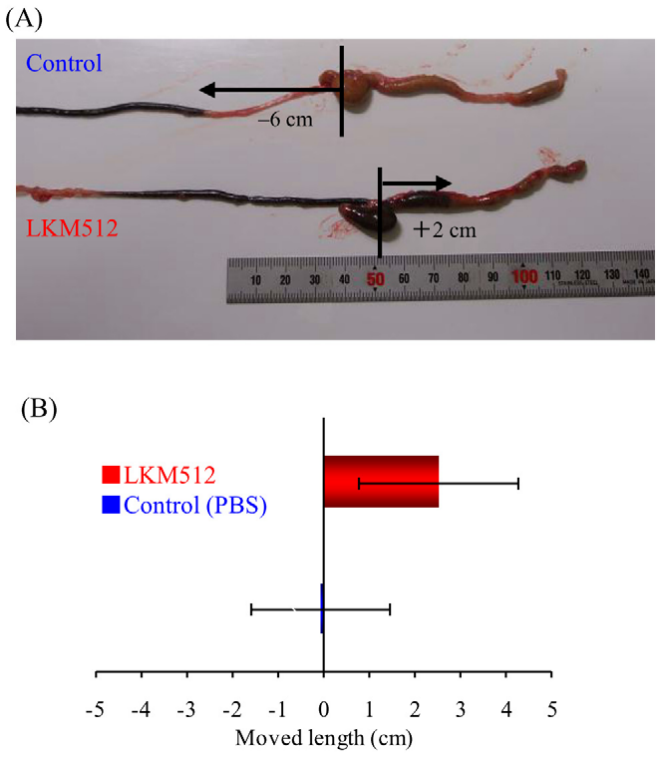
Effects of *B. animalis* subsp. *lactis* LKM512 on intestinal peristalsis of mice administered sennoside after kanamycin treatment. (A) Picture of the intestinal tract. The activity of intestinal peristalsis was expressed as the distance travelled by the charcoal powder from the end of the ileum (i.e. the base of the caecum or start of the colon) to the top of the charcoal powder, measured using a ruler. (B) Comparison of the length of charcoal powder from the end of the ileum. This value was significantly longer in LKM512-adminstered mice (n = 5) than in the control (n = 5) (*p*<0.05), modified Wilcoxon signed-rank test). Error bars show the standard error of the mean.

This study was performed using LKM512, because the laxative ability of LKM10070 was too strong in the sennoside hydrolysis test. An example of this test is shown in Figure 5. In almost all pairs, the distance travelled by the charcoal powder was greater in LKM512-treated mice than in the controls (n = 5), similar to that shown in Figure 5A. The distance travelled by the charcoal powder from the end of the ileum in LKM512-treated mice was significantly longer than that of control (*p*<0.05) (Figure 5B).

### Effect of LKM512 on intestinal microbiota after kanamycin treatment

**Figure 6:**
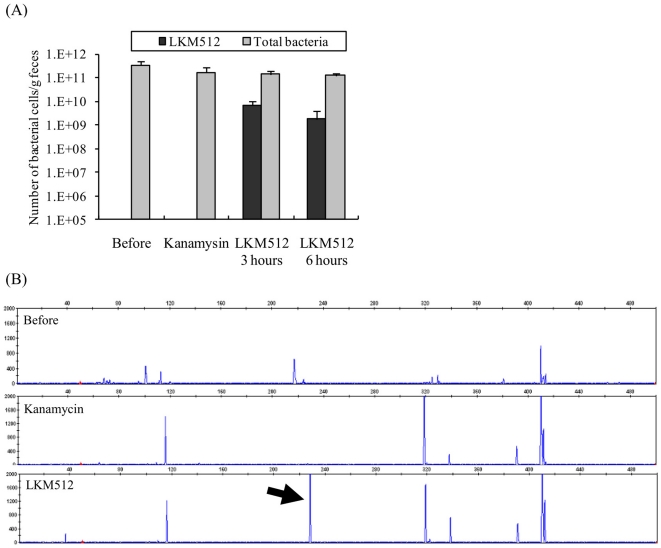
Effects of kanamycin and LKM512 administration after kanamycin treatment on intestinal microbiota. (A) Change in the number of total bacteria and *B. animalis* subsp. *lactis* LKM512 in feces. Since the detection limit of the standard curve is 10^3^ cells, bacterial counts below this limit were assigned a value of 10^3^ cells/g of feces. (B) Change in the T-RFLP profile after kanamycin treatment (middle) and LKM512 administration after kanamycin treatment (lower) on digestion with *Hae*III. Arrow indicates T-RF predicted to be derived from LKM512.

The intestinal bacterial count is shown in Figure 6A. The total bacterial count before the test and after kanamycin administration was 3.4±1.0×10^11^ cells/g of feces and 1.6±0.6×10^11^ cells/g of feces, respectively, showing a slight decrease in the intestinal microbial count upon kanamycin treatment. Furthermore, the total bacterial count did not recover until 6 hours after LKM512 administration. The number of *B. animalis* subsp. *lactis* (LKM512) before treatment was below the detection limit (10^3^ cells/g of feces). At 3 and 6 hours after LKM512 administration, these values were 6.8±1.8×10^9^ cells/g feces, and 1.8±1.3×10^9^ cells/g of feces, respectively.

Figure 6B shows the typical T-RFLP profiles prior to the treatment, after kanamycin treatment, and at 6 hours after LKM512 administration. Although 22 terminal restriction fragments (T-RFs) were obtained from the feces before the treatment, only 7 T-RFs were detected from the feces after kanamycin treatment, indicating that the diversity of the intestinal microbiota was reduced by kanamycin treatment. Although 230 bp T-RF, which was predicted to be derived from LKM512, was detected after LKM512 supplementation, the pattern of the other T-RFs detected was the same as that obtained in the T-RFLP after kanamycin treatment. Although there are individual differences in the T-RFLP profile of feces before treatment, the diversity of the intestinal microbiota was reduced by kanamycin treatment and the 230-bp T-RF was commonly detected after LKM512 supplementation.

## Discussion

Postoperative hospitalized patients or elderly individuals experience flaccid constipation, which is characterized by weak muscular activity (i.e. intestinal peristalsis) in the colon. Therefore, promotion of intestinal peristalsis using the sennoside-containing Kampo medicine *Daio* is an effective treatment. However, majority of such at-risk individuals frequently take antibiotics to prevent bacterial infection, consequently damaging the intestinal microbiota. [26] This explains why *daio* does not effectively treat constipation in many patients. Therefore, since our study involved experiments using mice treated with kanamycin, this study is very clinically relevant.

Two known pathways exist for sennoside metabolism by intestinal microbiota [14], [15], [27]. In the first pathway, sennosides A and B are first hydrolysed to sennidin A and sennidin B via sennidin A monoglucoside and sennidin B monoglucoside, respectively, by β-D-glucosidase; this is followed by reduction to produce rheinanthrone, a genuine purgative component. In the second pathway, sennosides are first reduced to 8-glucosyl-rheinanthrone and then hydrolysed by β-D-glucosidase(s) to produce rheinanthrone [14], [15], [27]. Intestinal bacterial species and strains which hydrolyse sennoside to sennidin, such as *Bifidobacterium* sp. strain SEN [13], *Clostridium sphenoides*, and *Bifidobacterium adolescentis* [14], and which hydrolysed sennidin to rheinanthrone, such as *C. sphenoides*, *Clostridium perfringens*, *B. adolescentis*, *Eubacterium limosum*, *Streptococcus intermedius* ( = *Peptostreptococcus intermedius*), and *Lactococcus lactis* subsp. *lactis* ( = *Lactobacillus xylosus*), were found [15]. From these reports, although sennoside appears to be hydrolysed to rheinanthrone in a stepwise manner by sennoside-hydrolysing bacteria and sennidin-reducing bacteria, *C. sphenoides* and *B. adolescentis* seem to have the ability to hydrolyse sennoside to rheinanthrone independently. *B. pseudocatenulatum* (LKM10070) and *B. animalis* subsp. *lactis* (LKM512) also are capable of hydrolysing sennoside to rheinanthrone.

No other study has tested bacterial strains involved in sennoside metabolism *in vivo*. These bacteria have to tolerate gastric juices and reach the lower intestinal tract after being ingested in order to be able to hydrolyse sennoside. LKM10070 and LKM512 have sufficient acid tolerance; in particular, we demonstrated that LKM512 exhibits potent acid tolerance [23] and the ability to adhere to intestinal mucin [24] and alter the human intestinal microbial populations [25]. In this study, *B. animalis* subsp. *lactis* (LKM512) was detected in the order of 10^10^ cells/g of feces, indicating that this strain was likely able to hydrolyze sennoside in the intestine of mice.

Oral administration of kanamycin resulted in a decrease in sennoside-hydrolysing bacteria already present in the intestine, since kanamycin results in a decrease in the intestinal microbial population and the disappearance of their diversity (Figure 6). However, because more than 10^11^ cells/g feces had survived after kanamycin treatment, the disappearance of the intestinal microbiota diversity probably contributes to the decrease in sennoside hydrolysis to a greater extent than does reduction of the intestinal bacterial population. The disappearance of diversity of intestinal microbiota implies downregulation of rheinanthrone production by the bacterial consortium that may contain sennoside-hydrolysing bacteria and sennidin-reducing bacteria. Facultative anaerobic bacteria, e.g., *Lactobacillus* or Enterobacteriaceae, which are among the commensal bacteria in the intestine of young mice, are the organisms that might have had an influence on sennoside hydrolysis since kanamycin does not have much effect on obligate anaerobic bacteria.

After oral administration of LKM512, we detected the T-RF which was predicted to be derived from this strain; however, the T-RF pattern was almost the same as that obtained after kanamycin treatment, indicating that the increased hydrolysis of kanamycin is most likely a direct result of the action of LKM512; however, there remains the possibility that administration of the strain has an indirect effect of causing alterations in the subdominant microbiota, which are undetectable by T-RF analysis. To the best of our knowledge, this is the first report demonstrating that orally administered bacterial strains promote intestinal peristalsis by producing rheinanthrone from sennoside in the intestine. *B. animalis* subsp. *lactis* LKM512 should be used in future clinical trials to treat constipation in postoperative hospitalized patients and elderly individuals.

Although positive effects were obtained by following the time schedule (Figure 1B), intestinal peristalsis were not promoted when LKM512 and sennoside were administered orally at the same time (data not shown). This indicates that sennoside passed through and was discharged from the colon without being hydrolysed before LKM512 could be activated in the intestine. For stable effects, further studies are required to determine an appropriate time of administration.

## Supporting Information

Table S1 Primer sets used for PCR.
(DOC)
