# Supplementary material for: Promotion of Intestinal Peristalsis by Bifidobacterium spp. Capable of Hydrolysing Sennosides in Mice
Source: PLoS One. 2012 Feb 22;7(2):e31700. doi: 10.1371/journal.pone.0031700 (PMC3284505; doi:10.1371/journal.pone.0031700)
Supplement: Table S1 — Primer sets used for PCR. (DOC) [file pone.0031700.s001.doc]

| **Supplementary Table S1.** Primer sets used for PCR | | |
| --- | --- | --- |
| Target | Sequence(5' to 3') | Annealing temp (ºC) |
| Universal primer | F: AGAGTTTGATCCTGGCTCAG | 50 |
|  | R: GGTTACCTTGTTACGACTT |  |
| *B. animalis* subsp. *lactis* | F: CCCTTTCCACGGGTCCC | 65 |
|  | R: AAGGGAAACCGTGTCTCCAC |  |
| Total bacteria | F: TCCTACGGGAGGCAGCAGT | 60 |
|  | R: GGACTACCAGGGTATCTAATCCTGTT |  |
